# Increased nociceptive sensitivity and nociceptin/orphanin FQ levels in a rat model of PTSD

**DOI:** 10.1186/1744-8069-8-76

**Published:** 2012-10-20

**Authors:** Yong Zhang, Priyam R Gandhi, Kelly M Standifer

**Affiliations:** 1 Department of Pharmaceutical Sciences, College of Pharmacy, University of Oklahoma Health Sciences Center, Oklahoma City, OK, 73117, USA; 2 Department of Cell Biology and Oklahoma Center for Neuroscience, College of Medicine, University of Oklahoma Health Sciences Center, Oklahoma City, OK, 73117, USA

**Keywords:** PTSD, Pain sensitivity, Nociceptin/Orphanin FQ, Allodynia, Elevated plus maze

## Abstract

**Background:**

Clinical studies indicate that post-traumatic stress disorder (PTSD) frequently shares co-morbidity with chronic pain. Although in animals acute stress-induced antinociception is well documented, the effect of PTSD-like stress on nociceptive sensitivity is unclear. Though a few studies measured nociceptive responses at a single time point, no studies have examined changes in nociceptive sensitivity over time following exposure to PTSD-like stress. Nociceptin/orphanin FQ (N/OFQ), an endogenous ligand for the N/OFQ peptide (NOP) receptor, modulates various biological functions in the central nervous system that are affected by PTSD, including nociceptive sensitivity, stress and anxiety, learning and memory.

**Results:**

The present study examined thermal and mechanical nociceptive sensitivity in male Sprague Dawley rats between 7 and 28 days after single-prolonged stress (SPS), an established animal model for PTSD. Rat paw withdrawal thresholds (PWT) to von Frey and paw withdrawal latencies (PWL) to radiant heat stimuli, respectively, dramatically decreased as early as 7 days after initiation of SPS and lasted the length of the study, 28 days. In addition, N/OFQ levels increased in cerebrospinal fluid (CSF; on days 9, 14 and 28) and serum (day 28), while levels of circulating corticosterone (CORT) decreased 28 days after initiation of SPS. SPS exposure induced anxiety-like behavior and enhanced inhibition of the hypothalamo-pituitary-adrenal (HPA) axis, as previously reported for this model.

**Conclusions:**

Our results demonstrate that SPS induces the development of persistent mechanical allodynia and thermal hyperalgesia that is accompanied by increased N/OFQ content in the CSF, and eventually, in serum. These findings suggest a link between N/OFQ and the development of hyperalgesia and allodynia in a rat model of PTSD.

## Background

Numerous clinical studies reported co-occurrence of PTSD with chronic pain *for review see*[1], but the source of the pain is not always an obvious one. In one study of U.S. veterans, 66% of PTSD patients were diagnosed with chronic pain [2]. A recent investigation revealed 58.6% of 340 Operation Enduring Freedom/Operation Iraqi Freedom (OEF/OIF) veterans developed both chronic pain and PTSD [3]. Assessment of pain sensitivity in PTSD patients suggests that PTSD can be associated with both increases and decreases in experimental pain sensitivity [1,4]. Acute stress-induced antinociception is well known in animals, whereas chronic stress is often associated with hyperalgesia [5-8]. However, our understanding about chronic pain and PTSD, and the ability of PTSD to alter pain sensitivity is limited due to the difficulty of translation from animal models of PTSD to human studies. Studies specifically examining the temporal dynamics of sensitivity to nociceptive stimuli following exposure to a PTSD-inducing event are lacking, thus limiting our search for effective therapies for both pain and PTSD. SPS, an established animal model for PTSD [9,10], mimics some of the physiological and behavioral changes described in PTSD patients that includes enhanced negative feedback to the HPA axis, anxiety behavior and cognitive impairments [11-14]. More importantly, SPS produces a PTSD-like state without subjecting animals to foot shock, thus facilitating assessment of nociception by paw withdrawal methods. Therefore, the first goal of this study was to determine if subjecting rats to PTSD-like conditions using the SPS model alters nociceptive sensitivity to mechanical and thermal stimuli over time.

Nociceptin/orphanin FQ (N/OFQ) [15,16] an endogenous ligand for the N/OFQ peptide (NOP) receptor, modulates various biological functions in the CNS, including nociceptive sensitivity, stress, anxiety, learning, memory and cytokine release [17]; all of which are affected by PTSD. Evidence from several studies suggests that the N/OFQ-NOP receptor system plays an important role in stress-related behaviors and activation of the HPA axis. Intracerebroventricular (icv) injection of N/OFQ elevates circulating adrenocorticotropic hormone (ACTH) and CORT levels in unstressed and mildly stressed rats, as well as produces anxiogenic effects [18-20]. However, anxiolytic effects following icv injections of N/OFQ also have been reported [21-24]. Thus, the role of N/OFQ in anxiety and in chronic, severe stress is still unclear. Numerous studies also have revealed a role for N/OFQ in pain modulation. In contrast to its antinociceptive properties upon spinal administration [25-27], supraspinal N/OFQ produces hyperalgesia in rats [25,28-30]. Elevated N/OFQ levels in serum were observed in patients with acute and chronic pain [31], but decreased N/OFQ was noted in CSF of patients receiving intrathecal morphine for pain [32], and in serum of patients suffering from fibromyalgia, cluster headache and migraine [33-35]. CSF and/or serum levels of N/OFQ also were elevated in animal models of chronic neuropathic pain [36-38]. Further, NOP antagonists block inflammatory and neuropathic pain [39-41]. Therefore, the second goal of this study was to determine if changes in nociceptive sensitivity are accompanied by changes in levels of N/OFQ. Expression of anxiety and evidence of enhanced HPA axis feedback were assessed to verify the validity of the SPS model in our hands. Results from this study indicate that exposure to a severe stressor can produce mechanical allodynia and thermal hyperalgesia within 7 days, that is sustained for at least another three weeks and is accompanied by increased N/OFQ.

## Results

### SPS produced anxiety-like behavior and enhanced negative feedback of the HPA axis

**Figure 1:**
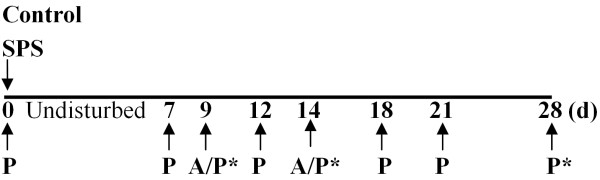
**Experimental paradigm.** Rats were assessed for baseline pain thresholds prior to SPS initiation at day 0. After 7 days of isolation, pain sensitivity (P) was monitored by assessment of nociceptive responses to mechanical and thermal stimuli on days 7, 9, 12, 14, 18, 21 and 28. Anxiety-like behavior (A) was tested by EPM on day 9 or 14, but not necessarily in rats that were euthanized on those days. Rats were euthanized (*) at day 9, 15 or 28. Sera and CSF samples were taken immediately for further analysis.

**Figure 2:**
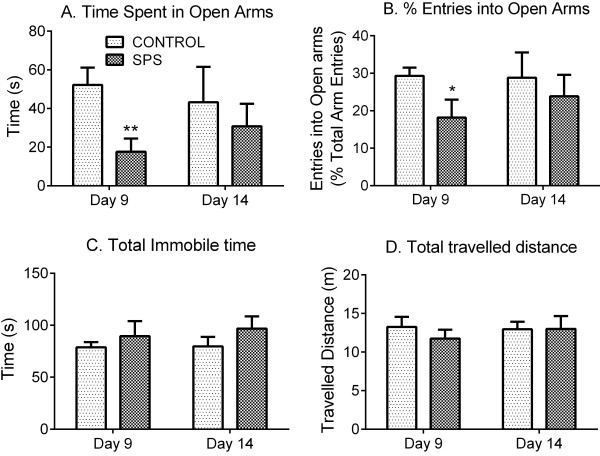
**SPS produced anxiety-like behavior in EPM test.** Two sets of rats were tested for 5 min duration on EPM at day 9 (control: n=12, SPS: n=11) or 14 (control: n=8, SPS: n=7) after SPS. At day 9 SPS-exposed rats spent significantly less time in open arms (A. **p<0.01) and made fewer entries into open arms (**B.** *p<0.05), consistent with anxiety-like behavior. No difference between groups was noted at day 14 after SPS. No significant difference in total immobile time (**C**) or distance travelled (**D**) between control and SPS rats was observed at either time point. Statistical analysis was carried out by unpaired Student’s t-test. Data are presented as mean ± SEM.

The SPS paradigm is a sequential exposure to four different stressors as described in methods: restraint, forced swim, exposure to diethyl ether until unconscious and social isolation for 7 days [9,10]. The complete experimental protocol is illustrated in Figure 1. To confirm the validity of the SPS model in our hands, the assessment of two symptoms of PTSD that are modeled by SPS was made [9,14]: the appearance of anxiety-like behavior and enhanced negative feedback of the HPA axis. The appearance of anxiety symptoms was examined in control rats and rats subjected to SPS using the Elevated plus maze (EPM) test (Figure 2). Student’s *t*-test revealed that 9 days post-SPS, rats spent significantly less time in the open arms of the EPM (*p*=0.0064), and made fewer entries into open arms (*p*=0.0409) than control animals (Figure 2A, B; Control: n=12, SPS: n=11). These anxiety-like behaviors cannot be explained by reduced mobility since locomotor function was not impaired by SPS; both groups spent equivalent time immobile and travelled a comparable distance (Figure 2C, D). Fifteen different rats were tested at day 14 after SPS (Control: n=8, SPS: n=7). No statistical difference between groups was detected at this time point, though the SPS rats showed a tendency to spend less time in open arms and make fewer entries into open arms than controls.

**Figure 3:**
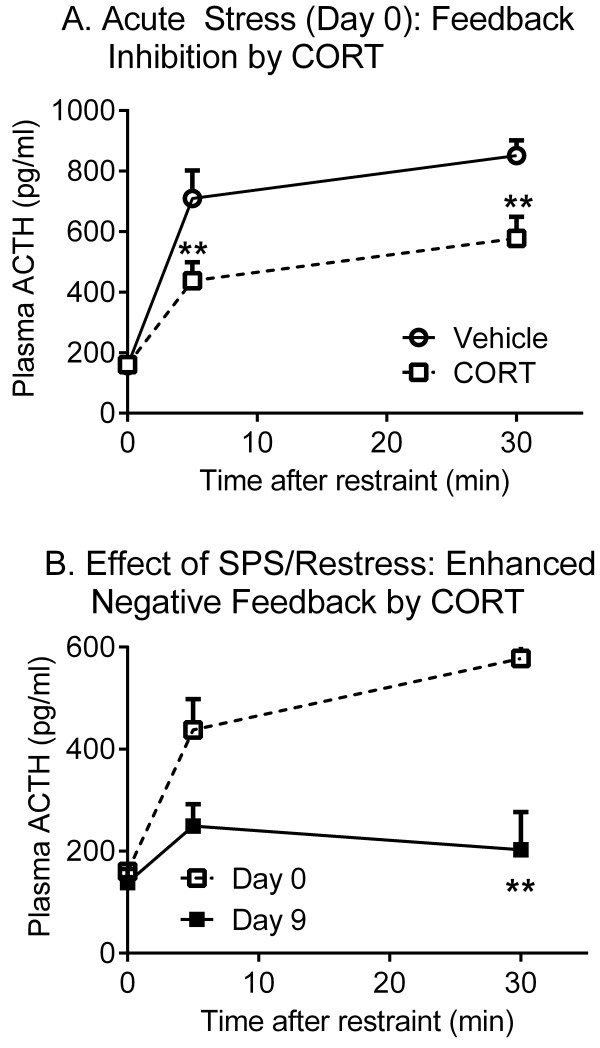
**Enhanced HPA axis inhibition after SPS.** (**A**) During the first 30 min of the 2 hr restraint phase of SPS at day 0, cortisol pretreated rats exhibited a significantly lower ACTH response to restraint compared to control rats that were subjected to SPS in the absence of cortisol injection as determined by repeated measures two-way ANOVA with Sidak’s multiple comparisons test [F(1, 10) = 9.136, *p*=0.0131] (Veh, vehicle injection. CORT, cortisol injection; n=6 for each group). (**B**) 9 days after exposure to SPS, cortisol pretreated rats exhibited greater inhibition of ACTH feedback compared to their response during the first restraint in day 0 [F(2, 10) = 6.272, *p*=0.0172] (n=6). Statistical analysis was carried out by two-way ANOVA with repeated measures (matching both factors) followed by Sidak’s multiple comparisons test (**p<0.005). Data are plotted as mean ± SEM.

A cortisol suppression test was conducted to further confirm that SPS produced enhanced negative feedback of the HPA axis in this study as was previously reported [9]. For this test, all rats (12) were subjected to SPS and blood was drawn at 0, 5 and 30 min after initiation of the first phase of SPS, the 2 hr restraint phase on day 0. Half of the rats received a cortisol injection prior to initiation of SPS as described in methods. Nine days later, animals were subjected to a 30 min restraint (re-stress) accompanied by a cortisol injection, and blood was drawn at 0, 5 and 30 min of the re-stress. On day 0, increased plasma ACTH levels were noted at 5 and 30 min after vehicle injection (Figure 3A; n=6). The increase in ACTH was significantly less in rats that received a cortisol injection prior to restraint (n=6) than in vehicle-treated controls (*p*<0.05 by Sidak’s multiple comparisons test). Two-way repeated measures ANOVA revealed a significant interaction between treatment and time [F(2, 20) = 5.422, *p*=0.0131], indicating that cortisol induced HPA axis negative feedback since ACTH levels were reduced in cortisol-treated rats compared to vehicle-treated controls (Figure 3A). There also was a significant effect of cortisol [F(1, 10) = 9.136, *p*=0.0128] and time [F(2, 20) = 71.37, *p*<0.0001] on ACTH levels. Nine days after exposure to SPS, cortisol pre-treated rats exhibited even lower ACTH response after re-stress compared to their day 0 responses (Figure 3B). Two-way repeated measures (by both factors) ANOVA revealed a significant interaction between day of treatment (acute vs. chronic stress/re-stress) and SPS [F(2, 10) = 6.272, *p*=0.0172], consistent with SPS enhancement of the negative feedback of HPA axis in this study, as was noted originally [9]. The effect of SPS [F(1, 5) = 8.767, *p*=0.0315] and time of collection of serum ACTH also were significant [F(2, 10) = 21.47, *p*=0.0002] (Figure 3B).

### SPS induced long-lasting allodynia and hyperalgesia

**Figure 4:**
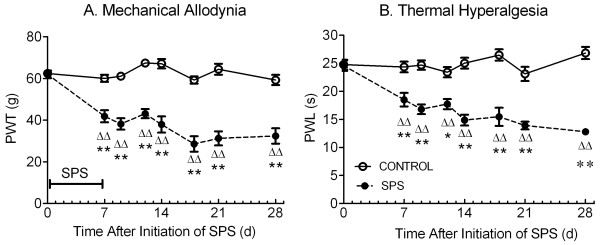
**Temporal Dynamics of SPS-induced mechanical allodynia (A) and thermal hyperalgesia (B).** Rat PWT to von Frey stimuli (control: n = 12–24, SPS: n = 11–22) and paw withdrawal latency (PWL) to radiant heat (control: n = 9–22, SPS: n = 9–20) dramatically decreased as early as 7 days after SPS and lasted the length of the study, 28 days. Sensitivity in each paw differed significantly from its pre-SPS threshold; ^ΔΔ^*p*<0.01) and from corresponding controls (*p<0.01; **p<0.001). Statistical analysis was carried out by two-way ANOVA followed by Sidak’s multiple comparisons test. Data plotted as mean ± SEM.

To determine if pain sensitivity was altered over 21 days following the 7 day isolation period of SPS, paw withdrawal thresholds (PWT) to mechanical nociceptive stimuli and paw withdrawal latencies (PWL) to thermal nociceptive stimuli were measured at least one hour before initiation of SPS at day 0 and at various time points between 7 and 28 days post-SPS as illustrated (Figure 1: control n=12-24: SPS n=11-22). Two-way ANOVA revealed a significant interaction between SPS treatment and time [F(7, 240) = 11.01, *p*<0.0001], indicating that changes in PWT differed in the two groups over time. Basal pain thresholds (day 0) to electronic von Frey stimuli were equivalent between groups. However, as early as day 7, SPS-exposed rats exhibited a decreased PWT in the right hind paw compared to their pre-SPS thresholds and to PWT in control rats. This allodynia was sustained throughout the remainder of the 28 day study. There also was a significant effect of SPS [F(1, 240) = 330.8, *p*<0.0001] and time [F(7, 240) = 12.07, *p*<0.0001] on PWT (Figure 4A). Specifically, mechanical allodynia appeared at day 7 of SPS. Post-hoc tests did not reveal differences in sensitivity within the control group over the 28 day period, indicating that the rats did not become sensitized to repeated assessments.

Similar results were noted with thermal stimuli as described above for mechanical stimuli. The interaction between time and SPS treatment was significant [F(7, 233) = 7.809, *p*<0.0001], indicating that changes in PWL differed over time between control and SPS rats. Two-way ANOVA revealed a significant effect of SPS [F(1, 233) = 197.4, *p*<0.0001] and time [F(7, 233) = 7.084, *p*<0.0001] on PWL to radiant heat. While control (n=12-24) and SPS-treated rats (n=11-22) had equivalent PWL to thermal stimuli at day 0, decreased PWL of the right hind paw to thermal stimuli also emerged at day 7 in SPS rats. This thermal hyperalgesia lasted through day 28 compared to pre-SPS threshold and to control rats (Figure 4B).

### N/OFQ levels in serum and CSF increased during SPS

**Figure 5:**
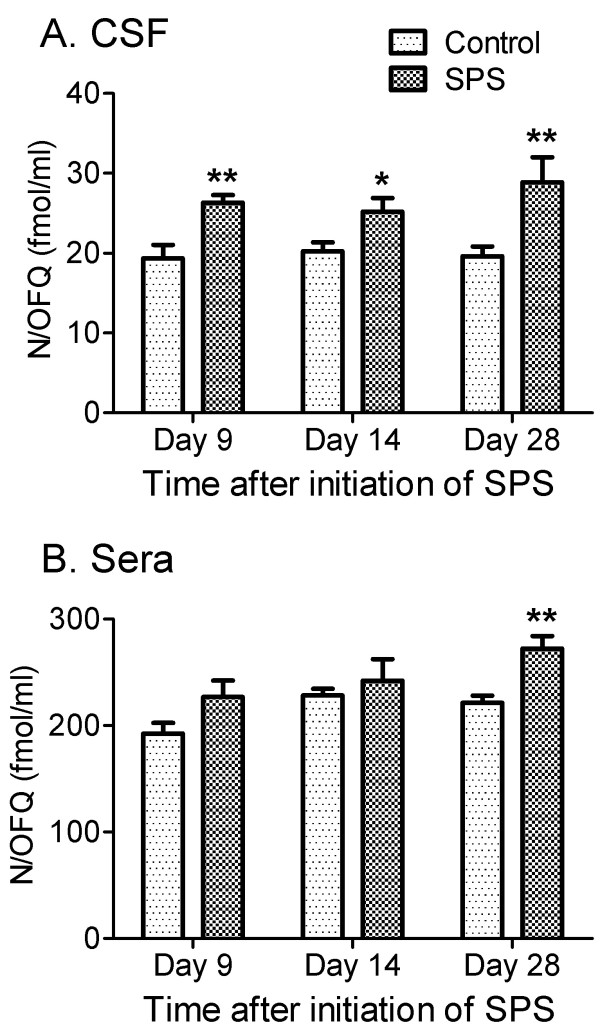
**SPS increased N/OFQ levels in CSF (A) and serum (B).** Serum and CSF were collected on day 9, 14 and 28 after SPS. N/OFQ level was elevated in CSF at day 9 (control: n = 4; SPS, n = 10. **p< 0.01), 14 (control: n = 8; SPS, n = 8. *p<0.05) and 28 (control: n=9; SPS, n = 7, **p<0.01). Serum N/OFQ level was elevated at day 28 (control: n = 12; SPS, n = 10. **p<0.01), but not day 9 (n=4 for control; n=10 for SPS) or 14 (n = 6/group). Statistical analysis was carried out by unpaired Student’s t-test. Data are plotted as mean ± SEM.

As indicated in the introduction, N/OFQ levels in serum and CSF of humans and rats increase with some types of acute and chronic pain, but decrease with others [31-38], suggesting that N/OFQ plays a complex role in pain processing. To determine if the appearance of allodynia and hyperalgesia are associated with changes in levels of N/OFQ in SPS, N/OFQ levels in serum and CSF were determined by RIA at day 9, 14 and 28 of SPS (Figure 5). Increased N/OFQ appeared earliest in CSF (Figure 5A) at day 9 (26.3 ± 1.0 fmol/ml in SPS rats (n=10) compared to 19.3 ± 1.7 fmol/ml in control (n=4, *p*=0.0026 by unpaired Student’s t-test). This increase was maintained at day 14 (SPS: 25.9 ± 1.8 fmol/ml; n=8; *p*=0.032) and at day 28 in SPS-treated rats (28.9 ± 3.12 fmol/ml, n=7; *p*=0.009), compared to controls (day 14: 20.2 ± 1.1, n=8; day 28: 19.6 ± 1.24 fmol/ml; n=9). An increase in serum N/OFQ levels in SPS-exposed rats was not noted until day 28 (272 ± 11.6 fmol/mL; n=10), which was significantly higher than levels in control rats (n=12, 221 ± 7 fmol/mL) as determined by unpaired Student’s t-test (Figure 5B, *p*=0.0008).

### Decreased corticosterone levels 28 days after initiation of SPS

**Figure 6:**
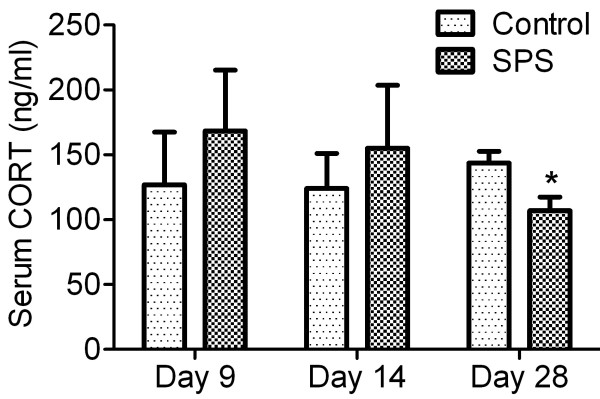
**SPS reduced long term serum CORT levels.** Serum was collected on day 9, 14 and 28 (control: n= 4, 4, 12, respectively; SPS: n=4, 4, 10, respectively) after initiation of SPS in 3 sets of rats. SPS exposure significantly reduced CORT levels at day 28, but not day 9 and 14, compared to control (*p<0.01) by unpaired Student’s t-test. Data plotted as mean ± SEM.

Since chronic hypocortisolism has been reported in many PTSD sufferers [42,43], the effect of SPS on the stress hormone, CORT, was measured in serum from SPS and control rats euthanized on day 9, 14 or 28 (Figure 6). Mean serum CORT levels were significantly decreased by day 28 of SPS (SPS: n=10, 107 ± 10 ng/ml) compared to control rats (control: n=12, 144 ± 9 ng/ml) as determined by unpaired Student’s t-test (*p*=0.014); No difference in serum CORT levels between SPS and control rats on day 9 or 14 was noted.

## Discussion

Considerable evidence indicates that acute stress, such as restraint [44-46] or swimming [47], induces antinociception. In contrast, chronic stress such as chronic restraint, rotational or swim stress is associated with allodynia or hyperalgesia [5-8,45,46]. However, no one has *ever* examined if subjection to PTSD-like conditions produces a painful condition or alters nociceptive sensitivity over time, despite the preponderance of reports citing increases or decreases in pain sensitivity in PTSD patients [1,4]. Therefore, we examined changes in nociceptive sensitivity to two different types of stimuli over a three week period following the 7 day SPS paradigm (Figure 4). Paw withdrawal thresholds to mechanical stimuli and paw withdrawal latency in response to thermal stimuli were reduced at the earliest time point tested, 7 days after initiation of SPS, compared to control rats and to pre-SPS thresholds in SPS rats. Moreover, the increased pain responses were maintained for at least 28 days after initiation of SPS. Correlational analysis of pain and anxiety data from SPS-treated rats on day 9 revealed that there was a significant correlation of allodynia and time spent in the open arms (*p*=0.04, Pearson’s r= 0.662). This correlation was not evident at day 14, when symptoms of anxiety seemed to dissipate. Since allodynia was still present, one might argue that the pain acted as a cue for anxiety, and that by day 14 and multiple pain assessments, the rats became conditioned to the pain cue.

Previous studies reported decreased [11,14] or unaltered [48] nociceptive sensitivity at a single time point in the SPS model. All measured electric shock-induced vocalization and jump thresholds in the flinch–jump test as a metric for pain sensitivity; in one of those studies the hot plate test also was employed [14]. Electric shock often is employed to produce fear or anxiety and is considered a nociceptive stimulus. That particular approach of assessing ‘pain’ sensitivity confounds the interpretation of data since SPS-treated rats may freeze in response to a foot shock conditioned stimulus, making it difficult to ascertain if lack of response is due to reduced nociceptive sensitivity or fear-induced immobility. The validity of our results is further supported by the fact that both mechanical allodynia and thermal hyperalgesia were observed throughout a three week period instead of a single time point.

Exposure to a high intensity stressor can induce long-lasting physiological and behavioral changes [5,6,12]. For example, 10–20 min of forced swimming for each of 3 days induced hyperalgesia to thermal and chemical stimuli 8 to 9 days after the last swim session [5]. In another case, somatic pain sensitization was noted 4 weeks after a single session of foot shocks [49]. The current study indicates that SPS systematically induces long lasting hyperalgesia to both thermal and mechanical stimuli, suggesting that PTSD-like conditions decrease the pain threshold and exaggerate nociceptive sensitivity.

Multiple lines of evidence suggest that endogenous N/OFQ levels are altered with pain symptoms in humans as well as in animal models. Serum N/OFQ levels were increased in acute and chronic pain patients, especially in those with chronic non-cancer pain compared to healthy controls [31]. However, plasma N/OFQ levels were reduced in patients suffering from fibromyalgia syndrome, cluster headache and migraine [33-35]. In animal models, N/OFQ levels are consistently increased with chronic pain conditions: nerve root compression (CSF; [36]), chronic constriction injury (serum; [37]), streptozocin-induced diabetic neuropathy (serum; [37]) and partial sciatic nerve ligation (serum; [38]). Endogenous N/OFQ also plays an important role in stress. Acute restraint stress reduced N/OFQ content in basal forebrain, but those neuronal stores were replenished within 24 h, implying that stress accelerated endogenous N/OFQ release and biosynthesis [50]. Acute restraint stress also enhanced N/OFQ expression in hippocampus [51]. Indeed, supraspinal N/OFQ blocks stress-induced analgesia [52]. Therefore, it was of particular interest to determine if chronic stress-induced hyperalgesia and allodynia from SPS were associated with changes in levels of N/OFQ in serum or CSF when compared to control rats. Concurrent with sustained mechanical allodynia and thermal hyperalgesia, the N/OFQ content in CSF was increased by 9 days post-SPS and remained elevated at days 14 and 28 days after initiation of SPS (Figure 5A). An increase in serum N/OFQ was not evident until day 28 (Figure 5B). The increase was more pronounced in CSF, in which N/OFQ increased 56% over levels in control rats on day 9. There may be multiple sources for the increased N/OFQ since it can be produced and released by neuronal, glial and immune cells (*for review, see*[17]). Pearson’s correlation analysis of SPS data indicates a significant correlation between N/OFQ levels in the CSF and allodynia at day 28 (*p*= 0.0109, r= 0.8701). Although it is not yet clear if elevated N/OFQ is a cause, consequence or coincidence of the increased nociceptive sensitivity, increased N/OFQ levels in serum and CSF at 28 day after initiation of SPS may reflect the broad spectrum of its roles in stress and maintenance of the hyperalgesia and allodynia during PTSD. Since this correlation was not found at day 9 or 14, it may suggest that CSF N/OFQ contributes to maintenance of a pain state that was initially a result of the stressor. Additional studies with NOP antagonist will be necessary to confirm if this is the case.

Rats exposed to the SPS model of PTSD exhibit enhanced HPA axis negative feedback in response to glucocorticoid administration [9], spend less time and make fewer entries into open arms of the EPM [14] and exhibit an exaggerated acoustic startle response [53], which are consistent with physiological and behavioral symptoms observed in PTSD patients [13]. The current study confirmed that SPS rats spent less time and made fewer entries into open arms of the EPM (Figure 2) and that the HPA axis negative feedback in response to cortisol administration was enhanced compared to control rats (Figure 3). These results validate the model of PTSD in our laboratory and indicate that allodynia and hyperalgesia were a direct result of SPS.

PTSD is associated with long-term dysregulation of the HPA axis and abnormal cortisol levels that increased in some studies of PTSD patients and decreased in others [54]. In animal models of PTSD, single exposure of an adult to a predator scent increased anxiety-like behavior AND plasma CORT. Exposure at both early and later life reduced CORT levels following the initial exposure, without increasing CORT levels upon re-exposure [55]. A PTSD model involving both repeated maternal separation and adult exposure to inescapable foot shock increased anxiety-like behavior and reduced basal CORT levels in plasma two months later [56]. Plasma CORT levels previously had only been assessed in the SPS model at early times points: CORT was elevated within one day of SPS initiation and returned to baseline levels at 7 days after SPS [45,57]. The long term effect of SPS on CORT levels has never been reported. Our results indicate that serum CORT levels dropped between 14 and 28 days after SPS (Figure 6), and suggest that SPS exposure induced long-term changes in CORT. These long-term changes parallel effects observed in larger, PTSD patient studies [39,40]. Interestingly, serum CORT levels correlate significantly with serum N/OFQ at day 28 (*p* = 0.0015, r = 0.8586). Though this relationship is not yet clear, it does suggest a relationship between N/OFQ and chronic stress.

## Conclusions

In conclusion, our novel findings demonstrate that animals subjected to PTSD-like conditions develop mechanical allodynia and thermal hyperalgesia that are apparent from the end of the SPS period (day 7) through at least the next 21 days (day 28). The allodynia and hyperalgesia in SPS rats is accompanied by elevated N/OFQ levels in CSF and serum. Though only correlative at this point, this new evidence suggests a role for N/OFQ in modulation of chronic stress-induced pain. The SPS-induced up-regulation of N/OFQ suggests a link between the N/OFQ-NOP receptor system and the development of hyperalgesia/allodynia induced by PTSD.

## Methods

### Animals

Adult male Sprague–Dawley rats (n=58) weighing 220–250 g at the initiation of SPS were obtained from Charles River Labs (Wilmington, MA). Animals were housed in the animal facility under a 12-h light: 12-h dark cycle (lights on at 0600 h) with free access to food and water. After arrival, rats were acclimated to the animal facility for 7-10 days before experiments were initiated. Experimental protocols were approved by the Institutional Animal Care and Use Committee of the University of Oklahoma Health Sciences Center and the US Army Medical Research and Materiel Command Animal Care and Use Review Office. Research was conducted in compliance with the Animal Welfare Act Regulations and other Federal Statutes relating to animals and experiments involving animals, and adheres to the principles set forth in the Guide for Care and Use of Laboratory Animals, National Research Council, 1996. All experiments conformed to the guidelines of the International Association for the Study of Pain. Efforts were made throughout experiments to minimize animal discomfort and reduce the number of animals used.

### SPS

Animals were randomly divided into control and SPS groups. The SPS procedure was followed as described [9,10] with modification. After 7 days of acclimatization, rats were exposed to complete restraint in disposable plastic holders for 2 hr, followed by grouped (4 rats) forced swimming for 20 min in a cylindrical plexiglass tank (46 cm tall × 20 cm in diameter) filled with 22°C water to a depth of 30 cm. After 15 min recovery, rats were exposed to diethyl ether until loss of consciousness and then left isolated and undisturbed for 7 days. SPS animals were individually housed throughout the 28 day study; control and SPS rats were assessed for nociceptive sensitivity over the next 21 days as described below.

### Elevated plus maze (EPM) test

Some rats were tested on the EPM on day 9 or 14 after SPS to determine the appearance of anxiety symptoms [58]. EPM tests were arranged between 0900 and 1030 h before the pain assessment. The plus maze consisted of two open (50 cm × 10 cm) and two closed (50 cm × 10 cm × 40 cm) arms elevated 40 cm above floor with average light levels 40–55 lux. Each rat was placed in the center of the apparatus facing the closed arms. The exposure was recorded with a video camera for 5 min and analyzed by Any-maze software (Stoelting Co., Wood Dale, IL). The percentage of open arm entries (number of entries into the open arm divided by total number of entries in both arms), the time spent in the open arms, the total distance traveled and total time spent immobile were calculated. None of the animals were tested on the EPM more than once.

### Pain sensitivity tests

This study examined changes in nociceptive responses to mechanical and thermal stimuli after SPS. A plantar analgesia meter (IITC Life Science Inc., Woodland Hills, CA) was utilized to measure PWL to an infrared light beam (thermal sensitivity) directed towards the right hind paw with the lamp set at 25% active intensity. Cut-off time was set at 30 sec to prevent tissue damage [59]. An Electronic von Frey anesthesiometer (IITC Life Science, Inc., Woodland Hills, CA) was utilized for mechanical nociception assessment. Rats were placed in clear plastic boxes with a wire mesh floor and paw withdrawal thresholds (PWT) were obtained from the mid-plantar aspect of the right hind paw. The responses to thermal and mechanical stimuli were tested 2 h apart. The average of 3 assessments spaced 5 min apart were compared between groups for each test. SPS was initiated at least one hour after baseline pain thresholds were assessed at day 0. After 7 days of isolation, pain sensitivity was assessed on days 7, 9, 12, 14, 18, 21 and 28. Rats were euthanized at day 9 (Control = 4; SPS= 4), 14 (Control = 8; SPS= 8) and 28 (Control = 12; SPS= 10). Serum and CSF samples were immediately taken for RIA analysis. The experimental paradigm is illustrated by the scheme in Figure 1.

### Cortisol suppression test

Twelve rats were randomly divided into two groups of 6 rats each. Immediately following placement of each rat into a disposable plastic holder in the first (restraint) phase of SPS on day 0, a small cut was made into the tail vein and blood was collected into an EDTA-containing microfuge tube to prevent coagulation and degradation of ACTH (time 0). One group of rats was injected subcutaneously with 3 mg/kg hydrocortisol, suspended in 10% ETOH + 0.9% NaCI (Sigma, St Louis, MO) immediately after acquisition of the baseline blood sample, and the other group received vehicle injection as a control. Additional blood samples were collected at 5 and 30 min of restraint. The SPS paradigm was continued as described (Figure 1) after the 30 min data collection point. Nine days after SPS, all rats were subjected to a cortisol injection (3 mg/kg, s.c.) plus re-stress restraint for 30 min. Tail nick samples were collected at 0, 5 and 30 min after restraint as noted for Day 0. SPS and tail nick was performed between 10:00–14:00 h; each sample was collected within 2 min. Whole blood (250~300 μl) was obtained at each time point and centrifuged at 4°C 5,000 × g for 5 min within 30 min of collection. Rats from this group were euthanized on day 9 after the last blood draw; CSF was collected for N/OFQ RIA from control rats (n=6); no pain assessments were made on these animals.

### Radioimmunoassay

At day 9, 14 or 28 rats were euthanized by Beuthanasia (Schering-Plough Animal Health, Union NJ). Blood was withdrawn from the heart with an 18-gauge needle (between 15:00 and 17:00 h), and maintained at room temperature for 30 min. Blood samples were centrifuged at 5,000 × g at 4°C for 5 min and the serum was collected and stored at −80°C. CSF from each rat was withdrawn by inserting a 26-gauge needle into the cysterna magna; CSF was immediately stored at −80°C. CORT levels in serum were determined by kit (MP Biomedicals, Orangeburg, NY) according to the manufacturer’s manual. The sensitivity of the assay was 25 ng/mL and non-specific binding was 2.6%. Total amount of CORT was calculated and expressed as ng/mL. N/OFQ content in sera and CSF was determined by kit (Phoenix Pharmaceuticals, Belmont, CA) according to the protocol suggested by manufacturer, and is presented as N/OFQ-IR. All samples and standards were assayed in duplicate. The sensitivity of the assay was 10 pg/mL and non-specific binding was 2.9%. There was no cross-reactivity with dynorphin A (1–17), enkephalin or β-endorphin. Total amount of N/OFQ was calculated and expressed as fmol/mL. The concentration of plasma ACTH was determined by RIA kit (MP Biomedicals, Orangeburg, NY) according to the manufacturer’s manual. The sensitivity of the assay was 10 pg/mL and non-specific binding was 4.7%. Total amount of ACTH was calculated and expressed as pg/mL.

### Data analysis

Data are expressed as mean ± S.E.M. Statistical comparisons of behavioral and neurochemical data were performed with unpaired Student’s t-test or two-way ANOVA (with or without repeated measures as noted in the text), followed by Sidak’s Multiple Comparisons post-tests using GraphPad Prism 6.0 software. Correlation matrices of pain thermal and mechanical pain assessments, anxiety parameters and N/OFQ and CORT levels were generated with data from SPS day 9, 14 and 28 rats presented herein; Pearson’s correlation coefficient (r) and p values determined by GraphPad Prism 6.0. Results were considered statistically significant if *p* < 0.05.

## Abbreviations

PTSD: Post-traumatic stress disorder; N/OFQ: Nociceptin/orphanin FQ; NOP receptor: N/OFQ peptide receptor; SPS: Single-prolonged stress; CORT: Corticosterone; CSF: Cerebrospinal fluid; OEF/OIF: Operation Enduring Freedom/Operation Iraqi Freedom; HPA: Hypothalamic–pituitary–adrenal; ACTH: Adrenocorticotropic hormone; EPM: Elevated plus maze; PWL: Paw withdrawal latency; PWT: Paw withdrawal threshold; RIA: Radioimmunoassay; IR: Immunoreactivity; icv: intracerebroventricular; sc: subcutaneous.

## Competing interests

The authors declare that they have no conflict of interest. The content of this paper does not necessarily reflect the position or the policy of the Government, and no official endorsement should be inferred.

## Authors’ contributions

The experiments were designed by YZ and KMS. YZ conducted the behavioral experiments and immunoassays, performed the statistical analysis and drafted the manuscript. PRG participated in the behavioral studies and edited the manuscript. KMS conceived of the study, performed the statistical analysis and drafted the manuscript. All authors read and approved the final manuscript.
